# The signaling pathways by which the Fas/FasL system accelerates oocyte aging

**DOI:** 10.18632/aging.100893

**Published:** 2016-02-09

**Authors:** Jiang Zhu, Fei-Hu Lin, Jie Zhang, Juan Lin, Hong Li, You-Wei Li, Xiu-Wen Tan, Jing-He Tan

**Affiliations:** ^1^ College of Life Science, Northeast Agricultural University, Harbin, 150030, P. R. China; ^2^ College of Animal Science and Veterinary Medicine, Shandong Agricultural University, Tai-an City 271018, P. R. China; ^3^ Department of Assisted Reproduction Shanghai Ninth People's Hospital, Shanghai Jiaotong University School of Medicine, 200011, P. R. China

**Keywords:** Fas/FasL, oocyte aging, phospholipase C, cytoplasmic Ca^2+^ rises, caspase-3

## Abstract

In spite of great efforts, the mechanisms for postovulatory oocyte aging are not fully understood. Although our previous work showed that the FasL/Fas signaling facilitated oocyte aging, the intra-oocyte signaling pathways are unknown. Furthermore, the mechanisms by which oxidative stress facilitates oocyte aging and the causal relationship between Ca^2+^ rises and caspase-3 activation and between the cell cycle and apoptosis during oocyte aging need detailed investigations. Our aim was to address these issues by studying the intra-oocyte signaling pathways for Fas/FasL to accelerate oocyte aging. The results indicated that sFasL released by cumulus cells activated Fas on the oocyte by increasing reactive oxygen species via activating NADPH oxidase. The activated Fas triggered Ca^2+^ release from the endoplasmic reticulum by activating phospholipase C-γ pathway and cytochrome c pathway. The cytoplasmic Ca^2+^ rises activated calcium/calmodulin-dependent protein kinase II (CaMKII) and caspase-3. While activated CaMKII increased oocyte susceptibility to activation by inactivating maturation-promoting factor (MPF) through cyclin B degradation, the activated caspase-3 facilitated further Ca^2+^ releasing that activates more caspase-3 leading to oocyte fragmentation. Furthermore, caspase-3 activation and fragmentation were prevented in oocytes with a high MPF activity, suggesting that an oocyte must be in interphase to undergo apoptosis.

## INTRODUCTION

If not fertilized in time following ovulation, the ovulated oocytes undergo a time-dependent process of aging both in vivo and in vitro. Because the post-ovulatory oocyte aging has marked detrimental effects on embryo development and offspring after natural mating or assisted reproduction [1], great efforts have been made to reveal its mechanisms. However, the molecular mechanism for oocyte aging is not fully understood.

Our recent work demonstrated that cumulus cells surrounding aging oocytes released sFasL in an apoptosis-related manner, and binding of the released sFasL to Fas receptors on the oocyte increased oocyte susceptibility to activating stimulus (STAS) and fragmentation [2]. However, the intra-oocyte signaling pathways by which the Fas/FasL system accelerates oocyte aging are unknown. In somatic cells, when Fas/FasL signaling is activated by the binding of FasL to Fas, the adaptor Fas-associated death domain protein (FADD) and pro-caspase-8 are recruited to form the death-induced signaling complex (DISC), which activates caspase-8 [3,4]. Caspase-8 cuts the Bcl-2 family member Bid to tBid, which promotes the release of pro-apoptotic factors such as cytochrome c [5]. The cytochrome c released from mitochondria binds with inositol trisphosphate receptor (IP_3_R) causing Ca^2+^ releasing [6]. On the other hand, Fas signaling elicits the production of inositol trisphosphate (IP_3_) through the activation of phospholipase C (PLC)-γ1, and IP_3_ binds to and activates IP_3_R that orchestrate the subsequent release of Ca^2+^ into the cytoplasm from endoplasmic reticulum stores [6,7]. Although it is known that the caspase-3-mediated truncation of IP_3_R1 enhances calcium release from the endoplasmic reticulum during oocyte aging [8,9], the causal relationship between caspase-3 activation and calcium releasing needs further investigations.

Calcium/calmodulin-dependent protein kinase II (CaMKII) has been implicated in oocyte activation [10]. Cytoplasmic calcium increases can activate CaMKII [11], which activates the anaphase promoting complexes/cyclosome (APC/C) by inhibiting Emi2 [12]. The activated APC/C targets proteins like cyclin B for degradation by the proteasome [13], leading to inactivation of the maturation-promoting factor (MPF). In addition, it has been shown in somatic cells that FasL triggers a rapid formation of reactive oxygen species (ROS), which activates Fas and induces apoptosis [14]. Further observations indicated that FasL promoted ROS generation by activating NADPH oxidases (NOX) [15,16]. Although recent studies have suggested that oxidative stress can decrease MPF and MAPK activities, impair calcium homoeostasis, induce mitochondrial dysfunction and directly damage multiple intracellular components such as lipids, proteins and DNA in the aging oocyte [1], the exact signaling pathways for oxidative stress to initiate these aberrations require detailed studies.

In brief, many studies have shown that aged oocytes have higher STAS but lower MPF and MAPK activity compared to newly ovulated oocytes [17]. Aged oocytes also suffered from higher oxidative stress, disturbed calcium homoeostasis, mitochondrial dysfunction and increased expression of caspase-3 [18]. Thus, we hypothesize that sFasL released by cumulus cells activates Fas on the oocyte by activating NOX and increasing ROS. The activated Fas promotes Ca^2+^ releasing by either the PLC-γ or the cytochrome c pathway. The cytoplasmic Ca^2+^ increases, on the one hand, activate CaMKII leading to cyclin B degradation and MPF inactivation, and on the other hand, activate caspase-3 resulting in oocyte fragmentation. The aim of the present study was to test our hypothesis and uncover the intra-oocyte molecular mechanism by which the Fas/FasL signaling accelerates oocyte aging. As the apparent phenomenon of postovulatory-aged oocytes include increased STAS [19] and cytoplasmic fragmentation [18], we used STAS and cytoplasmic fragmentation as markers for early and advanced oocyte aging, respectively.

## RESULTS

### The Fas/FasL system accelerates oocyte aging by decreasing the MPF activity

**Figure 1:**
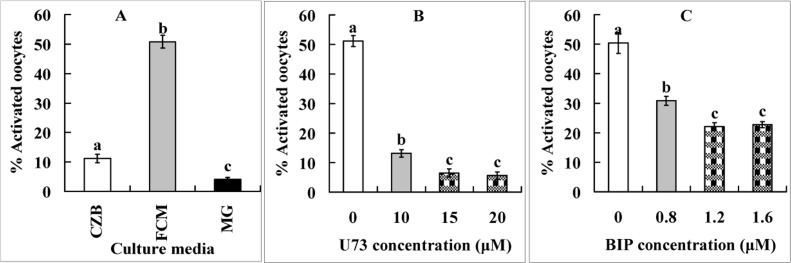
Ethanol activation rates after mouse oocytes collected 13 h post hCG were aged for 9 h in CZB, FCM or FCM containing 5-μM MG132 (MG) (Panel **A**) or different concentrations of PLC inhibitor U73122 (Panel **B**) or cytochrome c inhibitor B-IP3RCYT (Panel **C**). Each treatment was repeated 4 times with each replicate containing about 30 oocytes. a-c: Values without a common letter above their bars differ significantly (P < 0.05).

Newly ovulated oocytes were cultured for 9 h in CZB medium, FCM or FCM containing 5-μM MG132 before ethanol treatment for assessment of STAS. The ethanol activation rate was only 11% when oocytes were aged in CZB, but increased significantly to 50% after oocyte aging in FCM (Fig. 1A). When oocytes were aged in FCM containing MG132, however, activation rate decreased to less than 5%. The results suggested that Fas/FasL promoted oocyte aging by accelerating the degradation of cyclin B and inactivating MPF.

### The Fas/FasL system accelerates oocyte aging through PLC-γ and cytochrome c signaling pathways

Newly-ovulated oocytes were aged for 9 h in FCM containing different concentrations of PLC inhibitor U73122 or cytochrome c inhibitor B-IP3RCYT before ethanol treatment for STAS examination. Both inhibitors significantly reduced ethanol activation rates of oocytes (Fig. 1B and C). However, whereas the activation rate decreased dramatically to 6% when the PLC pathway was inhibited with U73122 (Fig. 1B), it was mildly reduced to about 22% when the cytochrome c pathway was inhibited with B-IP3RCYT (Fig. 1C). The results suggested that both the PLC-γ and the cytochrome c signaling pathways were involved in the Fas-acceleration of oocyte aging, but the PLC-γ pathway was more important than the cytochrome c pathway.

### The Fas/FasL signaling accelerates oocyte aging by altering the levels of cytoplasmic Ca^2+^ and Ca^2+^ stores

**Figure 2:**
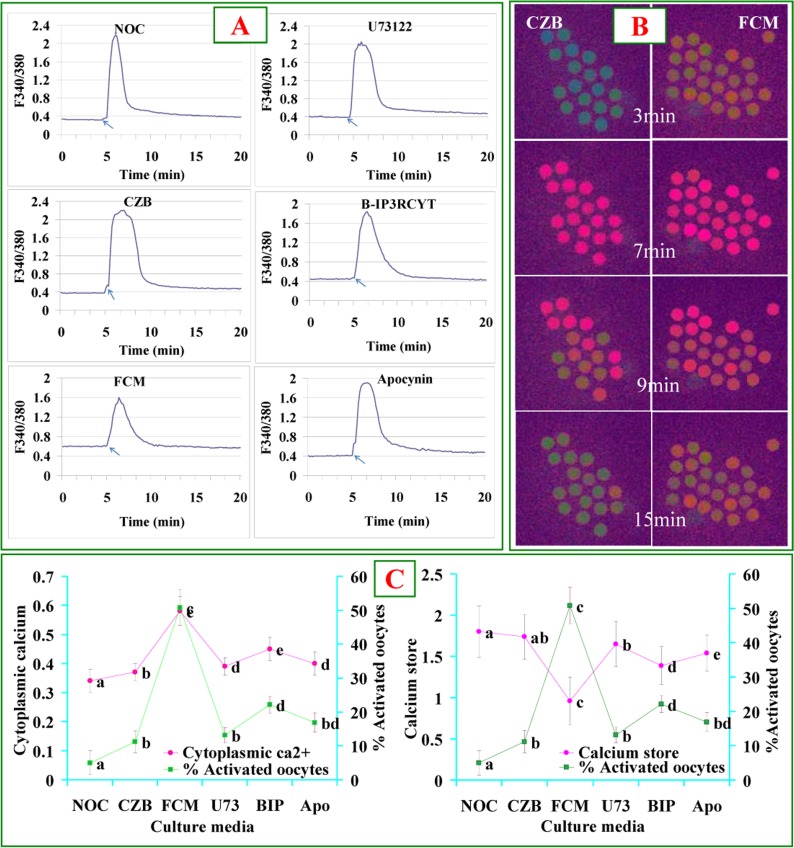
Levels of cytoplasmic calcium and calcium stores after oocytes were cultured for 9 h in different media. Panel A contains 6 graphs showing cytoplasmic calcium profiles in newly ovulated control (NOC) oocytes, and oocytes aged in CZB or FCM with U73122 (U73), B-IP3RCYT (BIP) or Apocynin (Apo), respectively. The arrows in graphs of Panel A indicate the time for ionomycin addition. Panel B contains Fura-2 images at different times of calcium measurement showing pseudo-colored F340/380 ratio after oocytes were cultured for 9 h in CZB or FCM. While the green color represents a low ratio, the red represents a high ratio of F340/380. Panel C consists of two graphs showing the correlation between oocyte ethanol activation rates and cytoplasmic calcium or calcium store, respectively, in oocytes aged in different media. To quantify calcium profiles, each treatment was repeated 3 times with each replicate containing about 20 oocytes. a-e: Values without a common letter differ significantly (P < 0.05).

Newly-ovulated oocytes were aged for 9 h in CZB or FCM containing different pathway regulators before Ca^2+^ measurement. While the level of cytoplasmic Ca^2+^ was higher, the Ca^2+^ store was lower significantly in oocytes aged in FCM than aged in CZB (Fig. 2). After oocytes were aged in FCM containing PLC inhibitor U73122, cytochrome c inhibitor B-IP3RCYT or NOX inhibitor apocynin, however, levels of cytoplasmic Ca^2+^ and Ca^2+^ store were close to that in oocytes aged in CZB. Our correlation analysis showed that whereas the cytoplasmic Ca^2+^ level was positively correlated (r=0.995, P<0.01), the Ca^2+^ store was negatively correlated (r=−0.987, P<0.01) with ethanol activation rates of oocytes (Fig. 2C). The results suggested that the Fas/FasL system accelerated oocyte aging by increasing the levels of cytoplasmic Ca^2+^.

### The Fas/FasL system facilitates oocyte aging by activating Ca^2+^/calmodulin-dependent protein kinase II (CaMKII) and caspase-3

**Figure 3:**
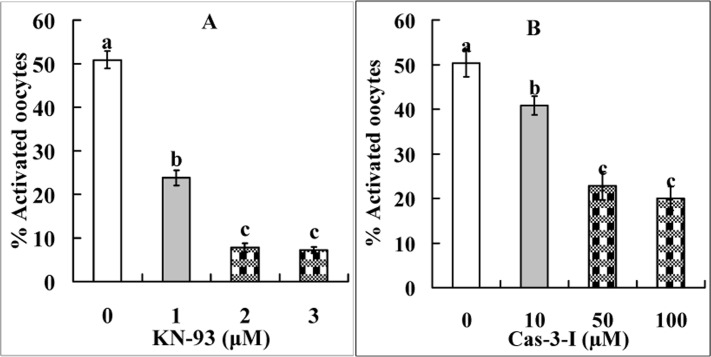
Ethanol activation rates after mouse oocytes collected 13 h post hCG were aged for 9 h in FCM containing different concentrations of CaMKII inhibitor KN-93 (Panel **A**) or caspase-3 inhibitor VII (Panel **B**). Each treatment was repeated 4 times with each replicate containing about 30 oocytes. a-c: Values without a common letter above their bars differ significantly (P < 0.05) within graphs.

Newly ovulated oocytes were aged in FCM containing different concentrations of CaMKII inhibitor KN-93 or caspase-3 inhibitor VII before ethanol treatment to assess STAS. Treatment with either KN-93 or caspase-3 inhibitor VII significantly decreased ethanol activation rates (Fig. 3). However, while the activation rate decreased to 7% when oocytes were aged in FCM containing 2 μM KN-93 (Fig. 3A), it reduced to over 20% (Fig. 3B) after treatment with caspase-3 inhibitor VII. The results suggested that (a) the Fas-induced ooplasmic Ca^2+^ rises facilitate oocyte aging by activating CaMKII and caspase-3 and (b) besides caspase-3, there are other factors that increase oocyte STAS.

### The Fas/FasL system increased oocyte fragmentation using similar signaling pathways as it used to increase STAS

**Figure 4:**
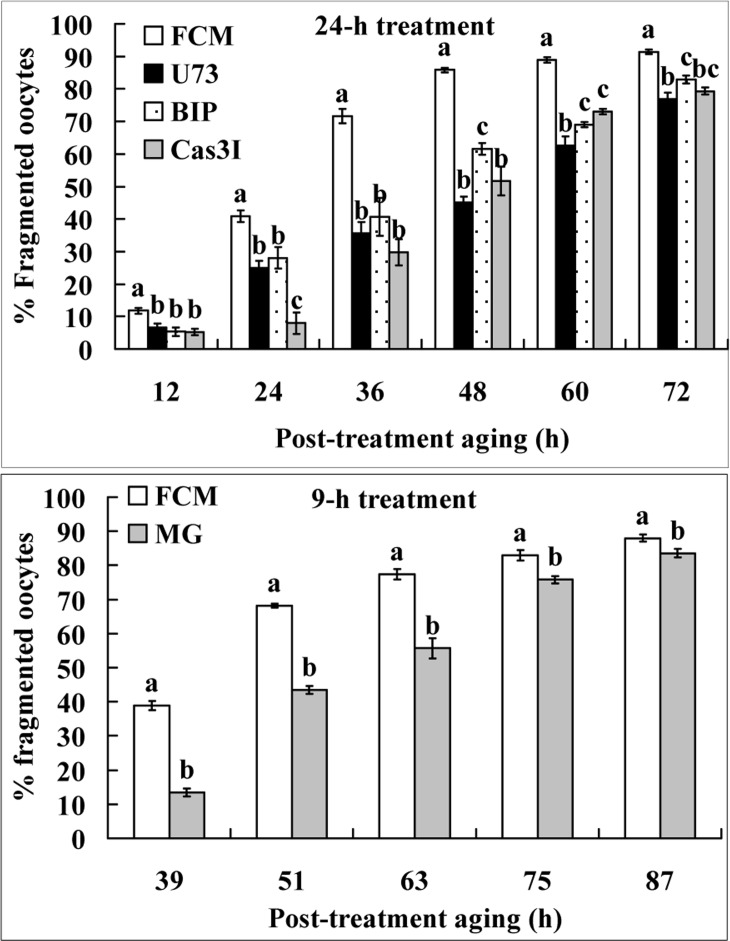
Fragmentation of aging oocytes. Newly-ovulated oocytes were either treated for 24 h in FCM alone or in FCM containing 10-μM U73122 (U73), 1.2-μM B-IP3RCYT (BIP) or 50-μM Caspase-3 inhibitor VII (Cas3I) (the 24-h treatment), or treated for 9 h with FCM alone or FCM containing 5-μM MG132 (the 9-h treatment), before post-treatment aging in CZB medium. At different times of the post-treatment aging, oocytes were observed for fragmentation. Each treatment was repeated 4 times with each replicate containing about 30 oocytes. a–c: Values without a common letter above their bars differ significantly (P<0.05) within time points of post-treatment aging.

Newly-ovulated oocytes were treated for 24 h in FCM alone or in FCM containing U73122, B-IP3RCYT or caspase-3 inhibitor VII before post-treatment aging in CZB. To study the effect of the cell cycle stage on oocyte fragmentation, some newly-ovulated oocytes were treated for 9 h in FCM alone or in FCM containing 5-μM MG132 before post-treatment aging in CZB. At different times of the post-treatment aging, oocytes were observed for fragmentation. Treatment with all the inhibitors significantly decreased the percentages of fragmented oocytes at different times of post-treatment aging (Fig. 4). The results suggested that (a) the Fas/FasL system used similar signaling pathways to increase oocyte fragmentation as it used to increase STAS, and (b) the maintenance of metaphase arrest with MG132 significantly inhibited fragmentation.

### The interrelationship between cytoplasmic Ca^2+^ rises and the caspae-3 activity during oocyte aging

**Figure 5:**
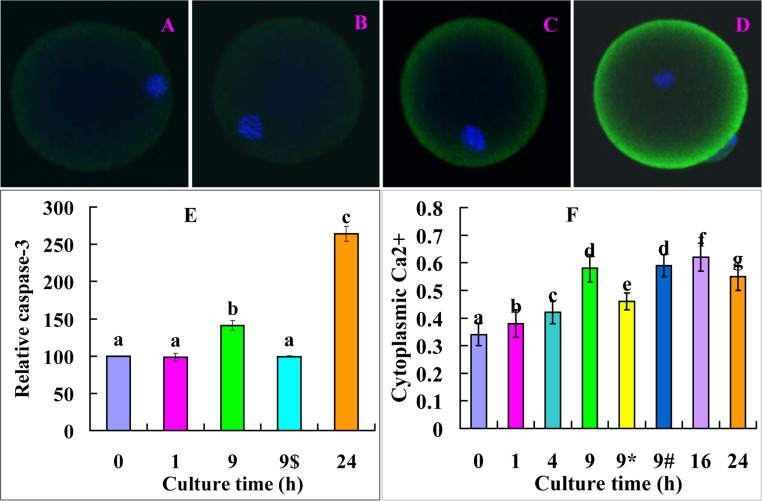
Levels of active caspase-3 and cytoplasmic calcium after oocytes were cultured in FCM for different times. A, B, C and D are confocal microscopic images (original magnification ×400) showing active caspase-3 distribution in oocytes aged in FCM for 0, 1, 9 and 24 h, respectively. E is a graph showing the quantification of active caspase-3 in oocytes aged in FCM for different times. 9$ indicates oocytes aged for 9 h in the presence of 5-μM MG132. Each treatment was repeated 3 times with each replicate containing 15-20 oocytes. F is a graph showing the levels of cytoplasmic calcium in oocytes aged in FCM for different times. 9* and 9# indicate oocytes aged for 9 h in the presence of caspase-3 inhibitor or 5-μM MG132, respectively. Each treatment was repeated 3 times with each replicate containing about 20 oocytes. a-g: values with different letters above their bars differ significantly (P<0.05) within graphs.

Newly-ovulated oocytes were cultured in FCM for different times before assessment for levels of active caspase-3 and cytoplasmic Ca^2+^. The level of active caspase-3 increased significantly from 1 to 9 h and reached the highest by 24 h of culture (Fig. 5A, B, C, D and E). The level of cytoplasmic Ca^2+^ increased significantly from 1 to 9 h and reached the highest by 16 h of culture (Fig. 5F). The results suggested that the elevation in cytoplasmic Ca^2+^ from 1 to 9 h of culture led to the significant increase in caspase-3 activity at 9 h of culture whereas the plateau of cytoplasmic Ca^2+^ observed between 9 and 16 h resulted in the dramatic increase in caspase-3 activity at 24 h of culture. On the other hand, the caspase-3 increase from 1 to 9 h evoked the big leap of cytoplasmic Ca^2+^ at 9 and 16 h of culture, because the Ca^2+^ level decreased significantly when oocytes were aged for 9 h in the presence of caspase-3 inhibitor VII (Fig. 5F, 9*). When oocytes were aged for 9 h in FCM containing MG132, while the level of cytoplasmic Ca^2+^ was similar to (Fig. 5F, 9#), the level of active caspase-3 (Fig. 5E, 9$) was lower significantly than that in oocytes aged in FCM alone, suggesting that MG132 did not affect cytoplasmic Ca^2+^ rises but inhibited caspase-3 activation during the Fas-facilitated oocyte aging.

### The FasL-induced oocyte aging is associated with increased oxidative stress

**Figure 6:**
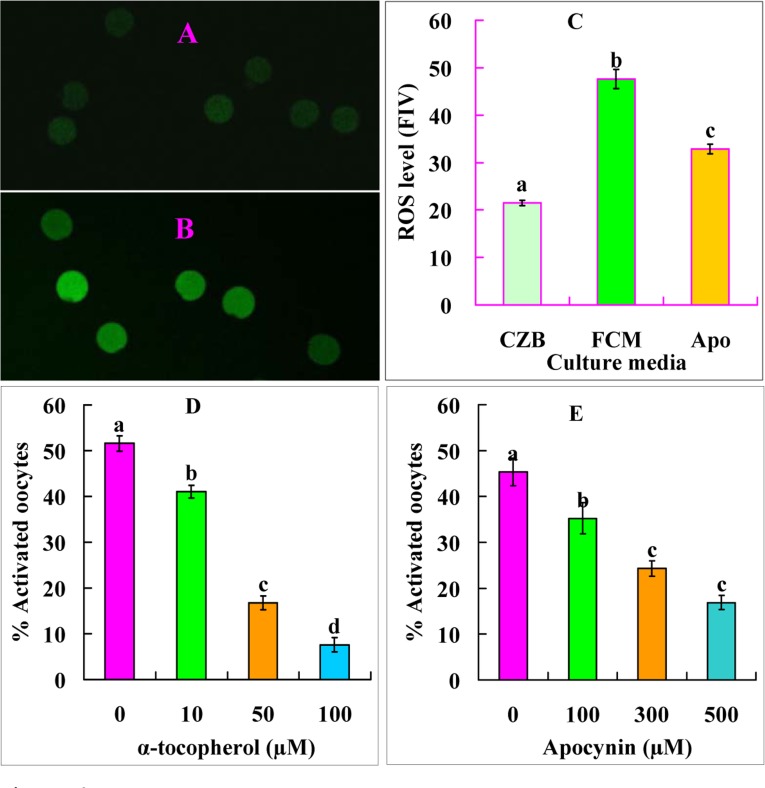
Levels of ROS and ethanol activation rates after mouse DOs were aged for 9 h in different media. A and B are confocal images showing the ROS fluorescence intensity of oocytes aged in CZB and FCM, respectively. C is a graph showing the quantification of ROS (fluorescence intensity value, FIV) in oocytes aged in CZB, FCM or FCM containing 500 μM apocynin (Apo). Each treatment was repeated five times with each replicate containing about 10 oocytes. D and E are graphs showing ethanol activation rates after oocytes were aged in FCM with different concentrations of α-tocopherol or apocynin, respectively. Each treatment was repeated 4 times with each replicate containing about 30 oocytes. a-d: Values with different letters above their bars differ significantly (P<0.05) within graphs.

Newly-ovulated oocytes were cultured in CZB or FCM for 9 h before assessment for ROS levels or ethanol treatment for activation. The ROS level was two-folds higher in oocytes aged in FCM than aged in CZB (Fig. 6A, B and C). Ethanol activation rates decreased significantly when oocytes were aged in FCM containing antioxidant α-tocopherol (Fig. 6D). The results suggested that FasL accelerated oocyte aging by inducing oxidative stress.

### The Fas/FasL system facilitates oocyte aging by activating NOX

Newly-ovulated oocytes were aged for 9 h in FCM containing different concentrations of NOX inhibitor apocynin before assessment for STAS. Ethanol activation rates decreased significantly with increasing concentrations of apocynin (Fig. 6E). In contrast, the ROS level was significantly lower in oocytes aged in FCM with than without apocynin (Fig. 6C). The results suggested that FasL facilitated oocyte aging by activating NOX.

### The inhibitors used in this study had no adverse effects on oocyte developmental potential

To rule out the possibility that the inhibitors we used had performed by their adverse effects (toxicity), effects of treatment with different inhibitors on oocyte developmental potential were examined. Newly-ovulated oocytes were cultured for 9 h in CZB with different inhibitors before Sr^2+^ activation for embryo development. The results showed that blastocyst percentages did not differ between oocytes cultured in CZB alone and oocytes cultured with different inhibitors (Table 1), suggesting that the inhibitors we used had no adverse effects on oocytes when used at the optimal concentrations to inhibit oocyte aging.

**Table 1:** Development of Sr2+ activated embryos after newly‐ovulated mouse oocytes were cultured for 9 h inCZB with different inhibitors

Inhibitors	Concentration (μM)	Oocytes cultured	% 4-cells/activated oocytes	% Blastocysts/4-cells
CZB control	0	118	95.4±0.9^a^	40.8±3.4^a^
U73122	10	120	93.7±0.9^a^	43.0±2.5^a^
B-IP3RCYT	1.2	109	90.7±2.6^a^	40.0±3.0^a^
KN-93	2	118	92.9±1.3^a^	41.2±1.5^a^
Cas-3-I VII	50	130	94.2±1.6^a^	58.8±1.6^c^
Apocynin	500	124	92.5±1.6^a^	37.3±2.3^a^

Each treatment was repeated 4 times with each replicate containing about 30 oocytes. a-c: Values without a common letter in superscripts differ (P < 0.05) in the same column.

## DISCUSSION

**Figure 7:**
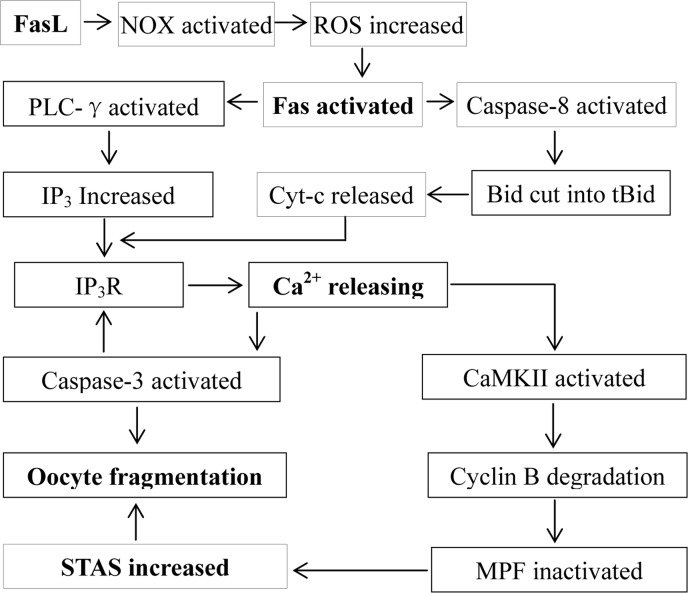
Possible pathways by which the FasL/Fas system facilitates oocyte aging. The sFasL released by cumulus cells activates Fas on the oocyte by activating NOX and increasing ROS. The activated Fas activates PLC-γ and caspase-8, which promote the production of IP_3_ and the release of cytochrome c, respectively. Both IP_3_ and cytochrome c interact with IP_3_R, triggering Ca^2+^ release from endoplasmic reticulum into the cytoplasm. The cytoplasmic Ca^2+^ rises activate CaMKII and caspase-3. While the activated CaMKII causes cyclin B degradation, MPF inactivation and an increase in STAS, the activated caspase-3 facilitates further calcium releasing by truncating IP_3_R, which activates more caspase-3 leading to oocyte fragmentation. On the other hand, the increase in STAS also facilitates oocyte fragmentation because a high MPF activity prevents caspase activation.

The present results suggested that the sFasL released by cumulus cells activates Fas on the oocyte by activating NOX and increasing ROS (Fig. 7). In somatic cells, numerous studies have demonstrated that ROS activates Fas and promotes cell apoptosis. For example, in CGD patients with a hereditary defect in ROS production, spontaneous cell death of neutrophils was significantly inhibited in vitro relative to normal neutrophils, and H_2_O_2_ increased both spontaneous apoptosis and Fas-mediated apoptosis of the CGD neutrophils [20]. Antioxidant vitamin C inhibits FAS-induced apoptosis in monocytes and U937 cells [21]. Furthermore, ROS plays an essential role in Fas-mediated apoptosome formation [22] and promotes Fas receptor clustering and cell death [23] in Jurkat cells. There are also many reports that FasL increases ROS production by activating NOX in somatic cells. In human B lymphoma cell lines BJAB and Ramos and the human T-cell leukemic cell line Jurkat, Fas induces ROS generation via stimulation of the NOX system [15]. In rat hepatocytes, FasL triggers a rapid formation of ROS as an upstream event of Fas activation by activating NOX isoforms [16]. In primary lung epithelial cells, NOX is a key source of ROS generation induced by FasL [14]. Expression of Nox genes has been observed in mouse oocytes [24], and inhibitors of NOX such as diphenyleneiodonium chloride [25] and apocynin [26] have been used to prevent meiotic maturation of mouse oocytes in the presence or absence of FSH.

The present results demonstrated that the activated Fas promoted Ca^2+^ releasing by activating PLC-γ (Fig. 7). In somatic cells, the activated Fas causes the production of IP_3_ by activating PLC-γ1, and the IP_3_ produced activates IP_3_R leading to the release of Ca^2+^ into the cytoplasm and to apoptosis [6,7]. In sea urchin eggs, PLC-γ plays a key role in several fertilization events including Ca^2+^ release and DNA synthesis [27]. In starfish, sperm-egg interaction causes egg activation by sequential activation of a Src-like kinase and PLC-γ [28]. In Xenopus eggs, fertilization requires Src-family PTK-dependent PLC-γ activity that acts upstream of the calcium-dependent signaling pathway [29]. In mammals, PLC-γ protein is present in mature mouse oocytes [30], and it is involved in oocyte activation induced by a truncated form of the C-kit tyrosine kinase present in spermatozoa [31]. Treating metaphase II mouse oocytes with U73122, the PLC inhibitor used in the present study, abolished Sr^2+^-induced Ca^2+^ increases [32]. While inhibiting PLC-γ completely prevented Ca^2+^ release in artificially activated mouse oocytes, it had no effect on Ca^2+^ release at fertilization [33]. Thus, the present results have provided further evidence that PLC-γ plays an important role in regulating aging and parthenogenetic activation of mammalian oocytes.

The present results also indicated that the activated Fas promoted Ca^2+^ releasing by inducing cytochrome c release from mitochondria (Fig. 7). In somatic cells, the activated Fas promotes the release of cytochrome c, which interacts with IP_3_R causing Ca^2+^ release and cell apoptosis [4,5,6]. In Xenopus, oocyte apoptosis induced by hyperosmotic shock is associated with cytochrome c release and caspase-3 activation [34], and the death process of unfertilized oocytes depends on both cytochrome c release and caspase activation [35]. During mammalian oogenesis, Fas ligand binding induces oocyte apoptosis by activating caspase 8; the activated caspase 8 then either activates executioner caspases directly, or trigger the cleavage of the bcl2 family member Bid, which causes cytochrome c release from mitochondria and activation of executioner caspases [36]. Furthermore, exposure of mouse zygotes to H_2_O_2_ induced cytochrome c release and apoptosis [37]. Thus, the present results have demonstrated, for the first time, that post-ovulatory aging increases STAS of mammalian oocytes by promoting the release of cytochrome c and Ca^2+^, which inactivates MPF by activating downstream proteins.

Our analysis on the interrelationship between cytoplasmic Ca^2+^ rises and the caspae-3 activity during oocyte aging revealed a reciprocal causation or mutual promotion effect between the two. Thus, the mild elevation in cytoplasmic Ca^2+^ observed from 1 to 9 h of culture in FCM caused an increase in the caspase-3 activity at 9 h of culture, which evoked a big leap of cytoplasmic Ca^2+^ at 9 and 16 h of culture, which, in turn, elicited a dramatic increase in the caspase-3 activity at 24 h of culture. Our recent studies have demonstrated that the increase in STAS in aged oocytes is associated with cytoplasmic Ca^2+^ rises in both mice and rats [38,39]. It was observed that oocyte aging altered the regulation of the intracellular Ca^2+^ concentration, and the resulting aberrant Ca^2+^ oscillations signaled apoptosis rather than activation in aged oocytes [18]. Furthermore, recent observations indicted that caspase-3 enhanced calcium release from the endoplasmic reticulum by cleaving IP_3_R1 during oocyte aging [8,9]. However, the causal relationship between caspase-3 activation and Ca^2+^ releasing during oocyte aging is unclear. Thus, the present results have provided the first evidence that an early mild Ca^2+^ release activates caspase-3, which causes further Ca^2+^ releasing; the elevated cytoplasmic Ca^2+^ activate more caspase-3 leading to oocyte fragmentation or apoptosis (Fig. 7).

In this study, after oocytes were aged for 9 h in FCM containing MG132, although the ethanol activation rate did not increase from that in newly-ovulated oocytes, the level of cytoplasmic Ca^2+^ elevated significantly to that in oocytes aged in FCM alone. However, treatment with MG132 significantly inhibited oocyte fragmentation and caspase-3 activation. Similarly, Choi also observed that DMSO inhibited fragmentation by maintaining a high MPF activity during in vitro culture of mouse oocytes [40]. Taken together, the results suggested that an apoptotic program had been initiated in the MG132-arrested metaphase oocytes, but it could not be executed until the oocytes were released from the metaphase arrest. Then, must a cell be in interphase to undergo apoptosis? It is known that differentiated cells in the body are arrested at G0 phase without cell-cycle progression. According to Tsuchiya et al. [41], when cellular injuries such as DNA damage or oxidative stress accumulate in proliferating cells, the cell-cycle is arrested in G1 or G2 phase before apoptosis induction. Furthermore, anti-cancer drugs inhibit cell proliferation and induce apoptosis often with a S-phase arrest [42,43].

Because changes in organelles or proteins during metaphase may prevent the correct execution of apoptosis [41], a special term ‘mitotic catastrophe’ has been proposed for cell death during mitosis [44]. In vitro reconstitution of apoptosis in cell-free extracts of Xenopus laevis eggs suggested that whereas extracts arrested in interphase were susceptible to an endogenous apoptotic program leading to caspase activation, extracts arrested in metaphase were not [45]. Further observations indicated that Mos/MEK/MAPK pathways active in metaphase-arrested eggs were responsible for rendering them refractory to apoptosis [46]. The metaphase-arrested extracts were competent to release cytochrome c, but they could not activate caspases. In this study, cytoplasmic Ca^2+^ increased to the same level in oocytes aging with or without MG132 to maintain a high MPF activity, but rates of fragmentation and the caspase-3 activity decreased significantly in the presence of MG132. Together, these data suggested that protein modification (phosphorylation) catalyzed by MAPK and/or MPF in metaphase may prevent caspase activation in spite of cytochrome c and Ca^2+^ releases. In fact, caspase-mediated cleavage of Xenopus Cdc25C took longer in metaphase than in interphase [41].

In summary, we have studied and revealed the signaling pathways by which the FasL/Fas system facilitates oocyte aging (Fig. 7). The new findings include, but are not limited to, the following. (a) The sFasL released by cumulus cells activated Fas on the oocyte by activating NOX and increasing ROS, revealing a new signaling pathway by which oxidative stress facilitates oocyte aging. (b) The mild cytoplasmic Ca^2+^ rises at the early stage of oocyte aging activated caspase-3, which facilitated further Ca^2+^ releasing that activates more caspase-3. The cycle between Ca^2+^ releasing and caspase-3 activation may continue until active caspase-3 reached a threshold level that triggered oocyte fragmentation at the late stage of oocyte aging. (c) The high MPF activity in metaphase oocytes prevented caspase activation in spite of cytochrome c and Ca^2+^ releases, suggesting that an oocyte must be in interphase to undergo apoptosis (fragmentation). (d) Although previous studies suggested that MAPK in metaphase-arrested Xenopus eggs might prevent caspase-3 activation with cytochrome c release [46], the level of cytoplasmic Ca^2+^ was not observed in that study. Thus, the present results that caspase-3 activation was prevented while Ca^2+^ release was unaffected in the metaphase-arrested mouse oocytes will help to specify the molecules through which MAPK/MPF inhibit caspase-3 activation. Taken together, the data are important not only for understanding the mechanisms for oocyte aging and Fas-induced apoptosis but also for understanding the functional relationship between the cell cycle and apoptosis.

## METHODS

The experimental procedures used for animal care and handling were approved by the Animal Care and Use Committee of the Shandong Agricultural University P. R. China (Permit number: SDAUA-2001-001). Chemicals and reagents used in the present study were purchased from Sigma Chemical Co. unless otherwise specified.

### Oocyte recovery

Mice of the Kunming breed were kept in a room with 14L: 10D cycles, with the dark period starting from 20:00. Female mice, 8–10 weeks after birth, were induced to superovulate with 10 IU equine chorionic gonadotropin (eCG, i.p.), followed 48 h later by 10 IU human chorionic gonadotropin (hCG, i.p.). Both eCG and hCG used in this study were from Ningbo Hormone Product Co., Ltd., China. The superovulated mice were killed 13 h after hCG injection, and the oviductal ampullae were broken to release cumulus-oocyte complexes. After being dispersed and washed three times in M2 medium, the cumulus-oocyte complexes were denuded of cumulus cells by pipetting with a thin pipette in a drop of M2 medium containing 0.1% hyaluronidase to prepare cumulus-denuded oocytes.

### Preparation of FasL-rich conditioned medium

FasL-rich conditioned medium (FCM) was prepared as described in our previous study [2]. Briefly, the cumulus cells produced during the preparation of cumulus-denuded oocytes were cultured for 24 h in regular CZB medium containing 200 μM H_2_O_2_. Then H_2_O_2_-treated cumulus cells were cultured in regular CZB medium (200 μl per well) for 24 h. At the end of culture, the medium was aspirated from the wells and centrifuged at 3000×g for 5 min to remove cells and debris. The FCM obtained was frozen at −80°C until use.

### In vitro aging of oocytes

For in vitro aging, oocytes were cultured for different times in regular CZB medium, FCM or FCM supplemented with different concentrations of inhibitors. To prepare stock solutions, MG132 (5 mM), U-73122 hydrate (2 mM), α-tocopherol (100 mM), Apocynin (100 mM, Santa Cruz), KN-93 (1 mM), Caspase-3 Inhibitor VII (20 mM, Merck Millipore) were dissolved in dimethyl sulfoxide; B-IP3RCYT (1.2 mM) was dissolved in PBS with 1% BSA. B-IP3RCYT was synthesized according to Boehning et al. [47], which is a cell permeant peptide encoding the cytochrome c binding domain of IP3R that can displace cytochrome c from IP3R. All the stock solutions were stored in aliquots at −20°C and diluted to desired concentrations with the aging medium immediately before use. The culture was conducted in wells (about 30 oocytes/well) of a 96-well culture plate containing 100 μl of medium, covered with mineral oil, at 37.5°C under 5% CO_2_ in humidified air.

### Activation of oocytes

In this study, ethanol stimulus was used to evaluate oocyte STAS, and the Sr^2+^ stimulus was used to assess the developmental potential of oocytes. For ethanol activation, oocytes were first treated with 5% (v/v) ethanol in M2 medium for 5 min at room temperature, then washed three times, and cultured in regular CZB medium containing 2 mM 6-dimethylaminopurine for 6 h at 37.5°C in a humidified atmosphere containing 5% CO_2_ in air. The activating medium used for Sr^2+^ activation was Ca^2+^-free CZB supplemented with 10 mM SrCl_2_ and 5 mg/ml cytochalasin B. After washing twice inM2 medium and once in the activating medium, the oocytes were incubated in the activating medium for 6 h. At the end of the activation culture, oocytes were examined under a microscope for activation. Only those oocytes that had one or two pronuclei, or two cells each having a nucleus, were considered activated.

### Embryo culture

Activated oocytes were cultured for 4 days in regular CZB medium (about 30 oocytes in a 100 ml drop) at 37.5°C under humidified atmosphere containing 5% CO_2_ in air. Glucose (5.55 mM) was added to CZB medium when the embryos were cultured beyond 3- or 4-cell stages.

### Measurement for cytoplasmic calcium and calcium stores

Oocytes were loaded with Ca^2+^ probe by incubating at room temperature for 20 min in HCZB medium (NaCl, 81.62 mM; KCl, 4.83 mM; KH_2_PO_4_, 1.18 mM; MgSO_4_, 1.18 mM; NaHCO_3_, 5.0 mM; HEPES, 20.0 mM; CaCl_2_•2H_2_O, 1.7 mM; sodium lactate, 31.3 mM; sodium pyruvate, 0.27 mM; EDTA, 0.11 mM; glutamine, 1 mM; bovine serum albumin, 5 g/L; penicillin, 0.06 g/L; streptomycin, 0.05 g/L) containing 1 μM Fura-2 AM and 0.02% pluronic F-127. Oocytes were transferred into a HCZB medium drop in Fluoro dish (FD35-100, World Precision Instruments) covered with mineral oil and observed with a Leica DMI6000 inverted microscope at 37.5°C. A Fura 2 fluorescence module was used for excitation, and a Leica LAS-AF calcium imaging module was used to calculate the F340/380 ratio, which represented the concentration of cytoplasmic calcium. The oocytes were monitored for 5 min to record the baseline F340/380 ratio before ionomycin stimulation to release Ca^2+^ into cytoplasm. For ionomycin stimulation, the drug was added to the HCZB medium drop to give a final concentration of 5 μM. Following ionomycin addition, the oocytes were monitored for 20 min to record the peak F340/380 ratio. The baseline F340/380 ratio represented the cytoplasmic calcium, and the difference between the peak and baseline F340/380 ratios represented the calcium stores of an oocyte.

### Assessment of oocyte fragmentation

Newly-ovulated oocytes were treated for 24 h in FCM alone or in FCM containing different inhibitors before post-treatment aging in CZB medium. At different times of post-treatment aging culture, oocytes were examined under a phase contrast microscope for morphological feature. Oocytes with a clear moderately granulate cytoplasm and an intact first polar body were considered un-fragmented, whereas oocytes with more than two asymmetric cells were considered fragmented [40].

### Immunofluorescence microscopy

Oocytes were washed 3 times in M2 medium between treatments. Oocytes were (i) fixed with 3.7% paraformaldehyde in PBS for 30 min, followed by treatment with 0.5% protease in M2 for several seconds to remove zona pellucida; (ii) permeabilized with 0.1% Triton X-100 in PBS at 37.5°C for 15 min; (iii) blocked in PBS containing 3% BSA at 37.5°C for 30 min; (iv) incubated with mouse-anti-active-caspase-3 (IgM, 1:100, Millpore) in 1% BSA in PBS at 37.5°C for 1 h; (v) incubated with FITC-conjugated donkey-anti-mouse IgM (1:200, Jackson ImmunoResearch) in 3% BSA in PBS at 37.5°C for 1 h; (vi) incubated for 5 min with 10 μg/ml Hoechst 33342 in M2 to stain chromatin. Negative control samples in which the primary antibody was omitted were also processed. The stained oocytes were mounted on glass slides and observed with a Leica laser scanning confocal microscope (TCS SP2). Blue diode (405 nm) and Argon (Ar; 488 nm) lasers were used to excite Hoechst and FITC, respectively. Fluorescence was detected with the following bandpass emission filters: 420-480 nm (Hoechst) and 492–520 nm (FITC), and the captured signals were recorded as blue and green, respectively. The relative content of active caspase-3 expression was quantified by measuring fluorescence intensities. For each experimental series, all high-resolution z-stack images were acquired with identical settings. The relative intensities were measured on the raw images using Image-Pro Plus software (Media Cybernetics Inc., Silver Spring, MD) under fixed thresholds across all slides.

### Assay for intraoocyte ROS

In order to quantify ROS in individual oocytes, intraoocyte H_2_O_2_ levels were measured using 2′,7′-dichlorodihydrofluorescein diacetate (DCHFDA) as described by Nasr-Esfahani et al.[48]. Stock solution of DCHFDA was prepared in dimethyl sulfoxide at 1 mM and stored in the dark at −20°C. The stock solution was diluted to 10 μM in M2 before use. Oocytes were stained for 10 min in DCHFDA solution at 37.5°C. After being washed thoroughly to remove the traces of the dye, at least 10 oocytes were placed on a slide, covered with a coverslip and observed under a Leica laser scanning confocal microscope. The fluorescence was obtained by excitation at 488 nm. Due to a rapid increment in the fluorescence intensity soon after staining, photographs were not taken until the fluorescence intensity reached a maximum and stable level (about 3 to 5 min after staining). Photographs were taken using fixed microscopic parameters, and the fluorescence intensity from each oocyte was analyzed using a Leica software.

### Data analysis

At least three replicates were performed for each treatment. Percentage data were arc sine transformed and analyzed with ANOVA; the Duncan multiple comparison test was used to locate differences. The software used was SPSS (Statistics Package for Social Science). Data are expressed as mean ± SE and P < 0.05 was considered significant.
